# Data on soil microbial carbon source utilization under different carbon input treatments in broadleaf and coniferous plantations

**DOI:** 10.1016/j.dib.2019.104434

**Published:** 2019-08-28

**Authors:** Yun Wang, Chi Zhang, Guangna Zhang, Xinli Wang, Bo Liu, Lizhi Wang, Yuan Gao, Xingyun Zhao, Heping Mei

**Affiliations:** a Shandong Provincial Key Laboratory of Water and Soil Conservation and Environmental Protection, College of Resources and Environment, Linyi University, Linyi, China; b College of Agriculture and Forestry Science, Linyi University, Linyi, China; c State-owned Tashan Forest Farm in Feixian County of Shandong Province, Linyi, China

**Keywords:** Carbon source utilization, Microbial functional metabolic diversity, Biolog Ecoplate™, Detritus input and removal treatments, Plantations

## Abstract

This article presents soil microbial carbon metabolism data under different detritus input and removal treatments (DIRT) in broadleaf and coniferous plantations in the Tashan Forests in Feixian County, Shandong Province, China (35°10′–36°00′N, 117°35′–118°20′E). The local annual air temperature is 13 °C, and the annual precipitation is 700 mm. The soil belongs to Phaeozems. The effects of DIRT on soil microbial carbon (C) metabolism in oak (*Quercus variabilis* Bl.) plantations and pine (*Pinus thunbergii* Parl.) plantations were assessed. There were five treatments for each plantation type, including: a control; doubling aboveground litter input; no aboveground litter input; no roots; and no detritus inputs. Soils were sampled after one year and nine months of DIRT. Soil microbial C metabolism was measured by EcoPlate™, which contained 31 different C substrates. The absorbance was measured with a micro-plate reader (Synergy H^1^, Biotek, Vermont, USA) at 590 nm every 12 h for 240 h. The data are based on 50 samples (two forest types × five C input treatments × five replicates); three replicates of the samples were taken. Interpretation of the data can be found in “Carbon input manipulations affecting microbial carbon metabolism in temperate forest soils – a comparative study between broadleaf and coniferous plantations” (Wang et al., 2019). The data can be used for studying the roles of aboveground and belowground inputs to soil C stabilization.

**Table:** Specifications Table

Subject	Ecology
Specific subject area	Soil biology and biochemistry; carbon source metabolism; detritus input and removal treatment; plantation
Type of data	Table
How data were acquired	Microbial C metabolic functions were measured using Biolog EcoPlate™ (Biolog Inc., Hayward, CA, USA). The absorbance was measured with a micro plate reader (Synergy H^1^, Biotek, Vermont, USA) at 590 nm every 12 h for 240 h.
Data format	Analyzed
Parameters for data collection	Soils were sampled after one year and nine months of C input type treatment in broadleaf and coniferous plantations. For each plantation type, the experiment was laid out in five replicated blocks. Each block was divided into five treatments. The five treatments included a control, doubling aboveground litter input, no aboveground litter input, no roots, and no detritus inputs.
Description of data collection	Soil microbial carbon metabolism was measured by Biolog Ecoplate™, whose absorbance was measured with a micro plate reader (Synergy H^1^, Biotek, Vermont, USA) at 590 nm every 12 h for 240 h.
Data source location	City/Town/Region: Feixian County/Shandong Province Country: China Latitude and longitude (and GPS coordinates) for collected samples/data: 35°10′–36°00′N, 117°35′–118°20′E
Data accessibility	With the article
Related research article	Author's name: Yun Wang, Chi Zhang, Guangna Zhang, Lizhi Wang, Yuan Gao, Xinli Wang, Bo Liu, Xingyun Zhao, Heping Mei Title: Carbon input manipulations affecting microbial carbon metabolism in temperate forest soils – a comparative study between broadleaf and coniferous plantations Journal: Geoderma https://doi.org/10.1016/j.geoderma.2019.113914

**Table:** 

**Value of the data** • The data provide insight into the detail values of soil microbial carbon source utilization profiles with Biolog EcoPlate™ under different detritus input and removal treatment in broadleaf and coniferous plantations. • The data will be of interest to soil scientists who study microbial carbon process, and also ecologists who study the roles of litter and roots in carbon metabolism in different forest types. • The data can be used for studying the relative role of aboveground and belowground plant inputs in soil carbon metabolism. • The data can be used for studying the difference in carbon metabolism of soil microorganisms in different forest types.

## 1 Data

The data derive from an investigation undertaken to see the influence of detritus input and removal treatments on carbon metabolism in broadleaf and coniferous plantations [1]. Soil microbial C metabolism was measured with Biolog EcoPlate™, which was incubated at 25 °C for 240 h. The absorbance was measured with a micro plate reader (Synergy H^1^, Biotek, Vermont, USA) at 590 nm every 12 h. The data included the average absorbance data of triplicates every 12 h for 240 h for 50 soil samples.

## 2 Experimental design, materials, and methods

### 2.1 Experimental design

The experiments reported herein were conducted in the Tashan Forests in the Feixian County of the Shandong Province, China (35°10′–36°00′N, 117°35′–118°20′E). The air temperature and annual precipitation in the region are 13 °C and 700 mm, respectively. The soil belongs to Kastanozems. The DIRT experiments were installed in Nov 2014, in oak (*Quercus variabilis* Bl.) and pine (*Pinus thunbergii* Parl.) plantations, both about 40-years old and with similar land-use history. The distance between the two types of plantations varied from tens of meters to several kilometers.

For each plantation type, the experiment was laid out in five replicated blocks. Each block (20 m × 30 m) was divided into five treated plots (1.5 m × 1.5 m) with five treatments randomly placed. The five treatments included control (CT), doubling aboveground litter input (DL), no aboveground litter input (NL), no roots (NR), and no detritus inputs (NI). The distance between plots varied from several meters to tens of meters. The buffer area between plots was at least 2 m × 2 m). For the DL, litter was doubled by adding litter removed from NL plots. For the NL, aboveground litter was removed with a 1-mm nylon mesh, and proportionately redistributed to the DL plots. For the NR, roots were excluded by inserting polyethylene sheeting in backfilled 50-cm deep trenches (just cut at the edge of plots). The understory vegetation including roots was removed manually. For the NI, litter and root inputs were excluded. There were no trees in any of the subplots. Practices demanded by treatments (i.e., detritus input and removal) were conducted once a month. The understory vegetation in the NR and NI plots was removed twice a month during the summer months.

### 2.2 Materials and methods

Soils were sampled on Aug 13, 2016, after one year and nine months of C input type treatment. Within each subplot, after removing surface litter, five soil cores (auger 3.6 cm in diameter) were driven into the soil to a depth of 10 cm to extract the soil samples, which were mixed to obtain one soil sample representative of each subplot. In all, 50 soil samples (two forest types × five treatments × five replicates) were collected and stored in an ice box before being transported to the laboratory. The samples were sieved through a 2-mm mesh, homogenized, and used in the analyses of microbial C metabolic functions.

Microbial C metabolic functions were measured using Biolog EcoPlate™ (Biolog Inc., Hayward, CA, USA) which contained 31 different C-substrates in a 96-well microplate. There are three replicates of each substrate and a no-C source control on each microplate. These 31 C-substrates were divided into six categories according to their chemical type, including carbohydrates, carboxylic acids, amino acids, amines/amides, polymers, and miscellaneous [2]. Field-moist samples equivalent to 10 g of dry soil were suspended in 95 mL of sterile 0.85% NaCl solution and agitated for 30 min before serially diluted 100 times. Then, 150 μL of each diluted soil suspension was inoculated into a well in the Biolog EcoPlate™, which was then incubated at 25 °C for 240 h. The absorbance was measured with a micro plate reader (Synergy H^1^, Biotek, Vermont, USA) at 590 nm every 12 h [3].
